# The Acute Effects of Simple Sugar Ingestion on Appetite, Gut-Derived Hormone Response, and Metabolic Markers in Men

**DOI:** 10.3390/nu9020135

**Published:** 2017-02-14

**Authors:** Adora M. W. Yau, John McLaughlin, William Gilmore, Ronald J. Maughan, Gethin H. Evans

**Affiliations:** 1 School of Healthcare Science, Manchester Metropolitan University, Manchester, Greater Manchester M1 5GD, UK; a.yau@mmu.ac.uk (A.M.W.Y.); b.gilmore@mmu.ac.uk or ws.gilmore@ulster.ac.uk (W.G.); 2 Institute of Inflammation and Repair, Faculty of Medical and Human Sciences, University of Manchester, Manchester, Greater Manchester M13 9PT, UK; john.mclaughlin@manchester.ac.uk; 3 School of Biomedical Sciences, Ulster University, Cromore Road, Coleraine, Co Londonderry BT52 1SA, UK; 4 School of Sport, Exercise and Health Sciences, Loughborough University, Loughborough, Leicestershire LE11 3TU, UK; R.J.Maughan@lboro.ac.uk

**Keywords:** glucose, fructose, sucrose, sugar ingestion, appetite, gut hormones, ghrelin, GLP-1, hepatic metabolism

## Abstract

This pilot study aimed to investigate the effect of simple sugar ingestion, in amounts typical of common ingestion, on appetite and the gut-derived hormone response. Seven healthy men ingested water (W) and equicaloric solutions containing 39.6 g glucose monohydrate (G), 36 g fructose (F), 36 g sucrose (S), and 19.8 g glucose monohydrate + 18 g fructose (C), in a randomised order. Serum concentrations of ghrelin, glucose dependent insulinotropic polypeptide (GIP), glucagon like peptide-1 (GLP-1), insulin, lactate, triglycerides, non-esterified fatty acids (NEFA), and d-3 hydroxybutyrate, were measured for 60 min. Appetite was measured using visual analogue scales (VAS). The ingestion of F and S resulted in a lower GIP incremental area under the curve (iAUC) compared to the ingestion of G (*p* < 0.05). No differences in the iAUC for GLP-1 or ghrelin were present between the trials, nor for insulin between the sugars. No differences in appetite ratings or hepatic metabolism measures were found, except for lactate, which was greater following the ingestion of F, S, and C, when compared to W and G (*p* < 0.05). The acute ingestion of typical amounts of fructose, in a variety of forms, results in marked differences in circulating GIP and lactate concentration, but no differences in appetite ratings, triglyceride concentration, indicative lipolysis, or NEFA metabolism, when compared to glucose.

## 1. Introduction

The ingestion of simple sugars has been the subject of much recent interest. In particular, the proportion of the daily energy intake from the ingestion of added fructose has rapidly increased, and this has been suggested to play a role in the development of metabolic syndrome and obesity [1,2]. Besides the ingestion of the fructose found naturally in fruits, fructose is typically ingested either as its component in sucrose or as high fructose corn syrup (commonly 55% fructose and 45% glucose). Fructose ingestion has been suggested to differentially alter feeding patterns to other simple sugars, leading to a resultant increase in body mass. One potential mechanism for the effect of fructose on feeding patterns is the effect that its ingestion may have on incretin and gut-derived hormones, which are known to influence subjective feelings of hunger.

Previous studies have shown that the acute ingestion of fructose increases blood glucose concentration [3], as well as the concentration of circulating insulin [3,4,5], though to a lesser extent than the ingestion of glucose. In addition, acute fructose ingestion has also been shown to increase circulating glucagon like peptide-1 (GLP-1) concentration [3] and to stimulate the secretion of leptin [5], though to a lesser extent than the ingestion of glucose. Furthermore, circulating levels of ghrelin following the ingestion of fructose are reported to be suppressed to a lesser extent than following the ingestion of glucose [6]. The effects of fructose ingestion on these circulating hormones, which are known to influence appetite, may therefore explain some of the reported relationships between the rise in dietary fructose ingestion and the increase in the prevalence of obesity.

While investigations in this area have demonstrated the effects of the acute ingestion of fructose on incretin and gut-derived hormone responses, a number of questions remain unanswered. Firstly, the majority of dietary fructose is ingested via sucrose or high fructose corn syrup. However, studies in this area have consistently investigated the effects of fructose alone. To date, and to the best of one’s knowledge, no studies have compared the effects of glucose, fructose, sucrose, and a combined glucose/fructose solution on incretin and gut-derived hormone responses. Secondly, according to the National Health and Nutrition Examination Survey (NHANES) data [7], the reported average daily fructose intake in the US is approximately 49 g. In the UK, the reported average daily intake of fructose is 39 g, with the recommended intake of free sugars being no more than 5% of the total energy intake [8]. For an adult aged between 19 and 74 years, this equates to approximately 24–35 g of free sugar, based on estimated average energy requirements [9]. However, many studies that have investigated the acute effects of fructose ingestion have used much higher quantities than this. 

The ingestion of large amounts of fructose in the diet is also being increasingly linked to non-alcoholic fatty liver disease (NAFLD), due to its differential and unfavourable metabolism in the liver, where it is considered to favour lipogenesis to a greater extent than glucose [10,11,12]. Studies indicate that short to moderate-term overfeeding with large amounts of fructose increases fasting and postprandial plasma triglyceride concentrations to a greater extent than glucose [5,6,13,14,15,16]. Studies have also shown that short to moderate term increases in fructose ingestion appears to favour the storage of fat to a greater extent than glucose as ingestion has been demonstrated to result in decreased lipolysis and metabolism of fatty acids, indicated by suppressed non-esterified fatty acid (NEFA) [6] and β-hydroxybutyrate [16] concentrations.

The effect of ingestion of an acute bolus of fructose and other simple sugars has also been documented with some conflicting findings. A mixed glucose and fructose solution with 45:55 g composition has been reported to elicit greater blood lactate and NEFA responses but no difference in triglyceride responses when compared to equivalent amounts of glucose alone [17]. On the other hand, serum triglyceride concentrations have been shown to be greater with mixed solutions of glucose and fructose of differing ratios compared to 85 g of glucose alone [18]. As with the studies investigating the effect of fructose ingestion on gut-derived appetite hormones, these acute ingestion studies on the hepatic processing of fructose have involved the ingestion of very high doses of sugars and the effect of a smaller quantity more reflective of a typical serving is unknown. The aim of this study was to examine the effect of simple sugar ingestion in more commonly ingested amounts on appetite, circulating gut hormone responses, and markers of hepatic metabolism.

## 2. Materials and Methods

### 2.1. Participants

Seven healthy men (mean ± standard deviation, age 25 ± 4 year, height 179 ± 8 cm, body mass 81.5 ± 12.3 kg, body mass index 25.5 ± 3.8 kg/m^2^, and body fat 21.0% ± 7.0%) volunteered to take part in this investigation. All participants were non-smokers and had no history of chronic gastrointestinal disease as determined via completion of a medical screening questionnaire. The participants provided written informed consent prior to participation and ethical approval was provided via the Institutional Ethical Advisory Committee (Reference Number: FAETC/10-11/67).

### 2.2. Experimental Procedure

Each participant completed five experimental trials with at least six days between trials. Experimental trials were completed in a single-blind randomised order and began at the same time each morning following the completion of pre-trial standardisation. Prior to the first experimental trial, participants were asked to record their dietary intake and physical activity but to refrain from the ingestion of alcohol and the participation of strenuous exercise. Participants were asked to replicate these dietary and physical activity patterns in the 24 h before each subsequent experimental trial. Experimental trials took place following an overnight fast from 2100 h, with the exception of the ingestion of 500 mL of water approximately 90 min before the arrival at the laboratory, in an attempt to ensure a consistent and adequate hydration status.

Following arrival to the laboratory, participants were asked to completely empty their bladder into a container, of which 5 mL was retained for later analysis. Following a measurement of body mass, participants lay in a semi-supine position while an intravenous cannula was inserted into an antecubital vein. This remained in place for the duration of the experimental trial and was kept patent by the infusion of saline after each blood sample collection. Participants completed a 10 cm visual analogue scale (VAS), assessing their level of hunger, fullness, and prospective food consumption. A baseline 5 mL blood sample was collected before participants ingested 595 mL of the test solution over a maximum period of two minutes. Test solutions contained water only (W), 39.6 g glucose monohydrate (G), 36 g fructose (F), 36 g sucrose (S), or 19.8 g glucose monohydrate + 18 g fructose (C). Test solutions were prepared to a volume of 600 mL and a 5 mL sample was retained for osmolality analysis. Participants remained in a semi-supine position for 60 min following drink ingestion. Further assessment of subjective feelings of appetite using a VAS were taken at 10 min intervals throughout this period and blood samples were collected 10, 20, 30, and 60 min after ingestion. Time-points for blood analysis were selected based on previous studies that showed that the ingestion of 75 g of fructose elicits peak concentrations of glucose and GLP-1 at approximately 30 min, before progressively declining to near baseline levels by 60 min [3]. Following the last sample collection, the cannula was removed and a second urine sample was collected before participants were allowed to leave the laboratory.

### 2.3. Sample Analysis

Urine, drink, and serum samples were analysed for osmolality by freezing point depression (Gonotec Osmomat 030, Gonotec, Berlin, Germany). Analysis was performed in duplicate. Upon collection of blood samples, 50 µL of Pefabloc (Roche Diagnostics Limited, Burgess Hill, UK) was immediately added to the blood to prevent the degradation of acylated ghrelin. Blood samples were centrifuged at 1500× *g* for 15 min and the serum was aliquoted then stored at −80 °C until analysis was performed. Serum glucose, lactate, triglyceride, NEFA, and d-3 hydroxybutyrate concentrations were determined in duplicate using a clinical chemistry analyser (Randox Daytona, Crumlin, UK), while serum fructose concentration was determined using a colorimetric assay (EnzyChrom™ EFRU-100; BioAssay Systems, Hayward, CA, USA). The circulating concentration of acylated ghrelin, insulin, and glucose dependent insulinotropic polypeptide (GIP) were determined using multiplex analysis (Luminex 200, Luminex Corporation, Austin, TX, USA), with kits purchased from Merck-Millipore (Milliplex MAP, Merck Millipore Ltd., Feltham, UK). The circulating concentrations of total GLP-1 were determined in duplicate, using Enzyme Linked Immunoassay (Merck Millipore Ltd., Feltham, UK).

### 2.4. Statistical Analysis

The incremental area under the curve (iAUC) for gut hormone and hepatic metabolism data was calculated using the trapezoid method. Differences in pre-trial body mass, pre-trial urine osmolality, drink osmolality, and gut hormone concentration iAUC were examined using one-way repeated analysis of variance (ANOVA). Significant *F*-tests were followed by Bonferroni-adjusted pairwise comparisons. Two-way repeated ANOVA were used to examine differences in urine osmolality, serum osmolality, blood glucose and fructose concentrations, gut hormone concentrations, hepatic metabolism concentrations, and subjective appetite VAS scores. Significant *F*-tests were followed with the appropriate paired Student’s *t*-tests or one-way repeated ANOVA and Bonferroni-adjusted pairwise comparisons. Sphericity for repeated measures was assessed, and where appropriate, Greenhouse-Geisser corrections were applied for epsilon <0.75 and the Huynh-Feldt correction was applied for less severe asphericity. All variables had full data sets with the exception of serum fructose for which eight samples (4.6% of total) were unable to be analysed and were therefore missing from the data analysis. Consequently, one data value (2.9% of total) was missing from the serum fructose iAUC data set. All data were analysed using SPSS Statistics for Windows (IBM, New York, NY, USA). Statistical significance was accepted at the 5% level and results were presented as means ± standard deviation (SD).

## 3. Results

### 3.1. Body Mass, Urine, and Drink Analysis

No change in body mass (Table 1) occurred during the study period (*p* = 0.638). Pre-trial urine volume and osmolality (Table 1) were not different between trials (*p* = 0.863 and *p* = 0.504, respectively). Post-trial urine volume was not different between trials (*p* = 0.231), and drinking resulted in reductions in urine osmolality in W, G, F, and S (*p* < 0.05), but not in C (*p* = 0.221).

The osmolality of ingested drinks were 13 ± 1, 370 ± 6, 368 ± 4, 204 ± 1, and 369 ± 4 mOsm/kg for W, G, F, S, and C, respectively. Drink osmolality for W was lower than all other solutions (*p* < 0.001) and drink osmolality for S was lower than G, F, and C (*p* < 0.001).

### 3.2. Serum Glucose, Fructose, and Lactate

**Figure 1:**
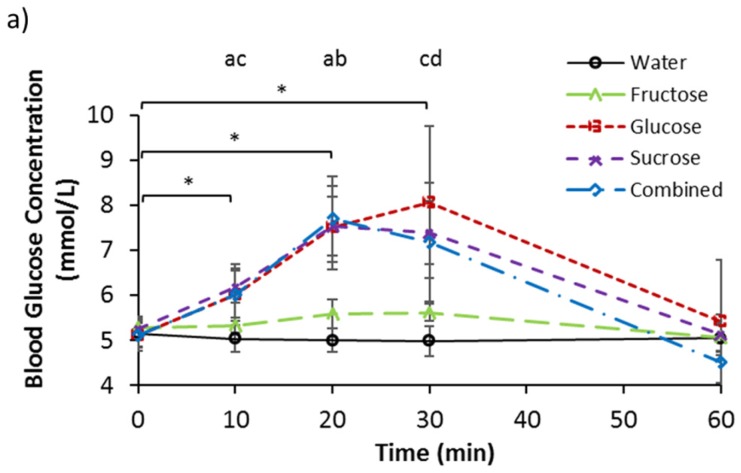

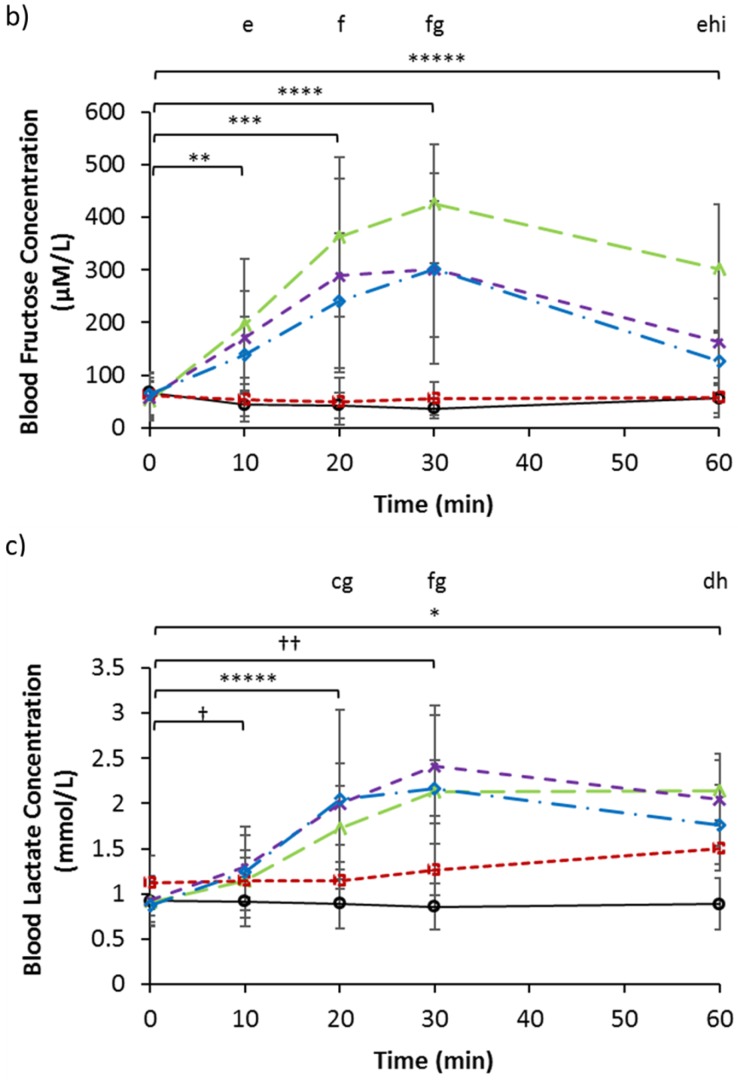
Serum (**a**) glucose (**b**) fructose and (**c**) lactate concentrations at baseline and following ingestion of 595 mL of water (W), 6% fructose (F), 6% glucose (G), 6% sucrose (S) and 6% combined glucose and fructose (C) solutions. ^a^ G, S, and C are greater than W; ^b^ G, S, and C are greater than F; ^c^ S is greater than F; ^d^ All carbohydrate trials are greater than W; ^e^ S is greater than G; ^f^ F is greater than W and G; ^g^ S is greater than W and G; ^h^ F is greater than G; ^I^ F is greater than C; * Increase from baseline for G, S, and C; ** Decrease from baseline for W; *** Increase from baseline for F; **** Decrease from baseline for W, and increase for F and S; ***** Increase from baseline for F and S; ^†^ Increase from baseline for S; ^††^ Increase from baseline for S and C. All *p* < 0.05. Values are mean ± standard deviation.

Baseline serum glucose concentrations (Table 2) were not different between trials (*p* = 0.288). Effects of trial (*p* < 0.001), time (*p* < 0.001), and interaction (*p* < 0.001), were present for serum glucose concentration (Figure 1a). Concentrations were elevated from pre-ingestion values at 10, 20, and 30 min after the ingestion of G, S, and C (*p* < 0.05). No difference was observed from baseline after the ingestion of W and F. At 10 min, blood glucose concentrations for G, S, and C, were greater than W (*p* < 0.05) and the concentration for S was greater than for F (*p* < 0.05). At 20 min after ingestion, blood glucose concentrations were greater for G, S, and C, than for W and F (*p* < 0.05). Furthermore, at 30 min after ingestion, blood glucose concentrations were greater for G, S, C, and F, compared to W (*p* < 0.05), while the concentration for S was greater than F (*p* < 0.05). Incremental AUC values for serum glucose concentration were −7.48 ± 12.17, 96.73 ± 55.64, 7.81 ± 9.78, 73.36 ± 24.39, and 66.61 ± 38.33 mmol/L 1 h, for W, G, F, S, and C, respectively. Trials G, S, and C were greater than W (*p* < 0.05), and S was greater than F (*p* = 0.005).

Baseline serum fructose concentrations (Table 2) were not different between trials (*p* = 0.912). Effects of trial (*p* < 0.001), time (*p* = 0.001), and interaction (*p* < 0.001), were present for the serum fructose concentration (Figure 1b). Concentrations were lower at 10 min (*p* = 0.032) and 30 min (*p* = 0.012) compared to pre-ingestion values for W. Serum fructose concentrations were greater than pre-ingestion values at 20, 30, and 60 min for F, and at 30 and 60 min for S (*p* < 0.05). At 10 min after ingestion, serum fructose concentration was greater for S compared to G (*p* = 0.036). At 20 min after ingestion, the concentration was greater for F compared to W (*p* = 0.043) and G (*p* = 0.038). At 30 min post ingestion, concentrations for F and S were greater than W and G (*p* < 0.05). At 60 min after ingestion, concentrations were greater for F and S compared to G (*p* < 0.05) and F was greater than C (*p* = 0.041). Incremental AUC values for the serum fructose concentration were −1194.12 ± 587.20, −451.19 ± 513.01, 15,780.52 ± 4156.57, 10,480.64 ± 4631.63, and 8788.90 ± 4665.14 µmol/L 1 h, for W, G, F, S, and C, respectively. Trials F, S, and C were greater than both W and G (*p* < 0.05).

Baseline serum lactate concentrations (Table 2) were not different between trials (*p* = 0.074). Effects of trial (*p* < 0.001), time (*p* < 0.001), and interaction (*p* < 0.001), were present for serum lactate concentration (Figure 1c). Concentrations were elevated from pre-ingestion values at all time-points for S (*p* < 0.05), and for F at 20, 30, and 60 min (*p* < 0.01). Elevations from baseline concentrations were observed for C at 30 (*p* = 0.043) and 60 min (*p* = 0.004), and for G at 60 min only. At 20 min after ingestion, concentrations were greater for S, compared to W, F, and G (*p* < 0.05), and at 30 min, concentrations were greater for S and F, compared to W and G (*p* < 0.05). At 60 min, the concentration for W was lower than all other trials (*p* < 0.05), and the concentration for F was greater than G (*p* < 0.05). Incremental AUC values were greater for F, S, and C compared to W and G (*p* < 0.05), with values 53.38 ± 6.32, 60.49 ± 18.45, 54.42 ± 27.10, −2.18 ± 8.21, and 8.88 ± 8.22 mmol/L 1 h, respectively.

### 3.3. Serum Insulin, GIP, GLP-1, and Ghrelin

**Figure 2:**
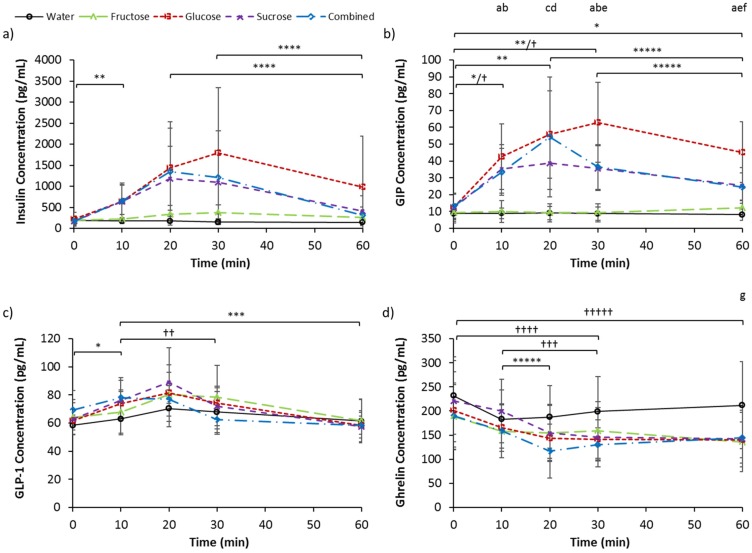
Serum (**a**) Insulin (**b**) glucose dependent insulinotropic polypeptide (GIP) (**c**) glucagon like peptide-1 (GLP-1) and (**d**) ghrelin concentrations at baseline and following ingestion of 595 mL of water (W), 6% fructose (F), 6% glucose (G), 6% sucrose (S) and 6% combined glucose and fructose (C) solutions. ^a^ W is less than G, S, and C; ^b^ Fructose is less than G, S, and C; ^c^ W is less than G and S; ^d^ F is less than G and S; ^e^ S is less than G; ^f^ C is less than G; ^g^ W is greater than F; * Increase from baseline for G; ** Increase from baseline for G and S; *** Decrease from time-point for G, S, and C; **** Decrease from time-point for G; ***** Decrease from time-point for S; ^†^ Increase from baseline for C; ^††^ Decrease from time-point for C; ^†††^ Decrease from time-point for C and S; ^††††^ Decrease from baseline for S and C; ^†††††^ Decrease from baseline for F and S. All *p* < 0.05. Values are mean ± standard deviation.

Baseline serum insulin concentrations (Table 2) were not different between trials (*p* = 0.587). Effects of trial (*p* = 0.032), time (*p* = 0.014), and interaction (*p* < 0.001), were present for serum insulin concentration (Figure 2a). Insulin concentrations were elevated from pre-ingestion values for G and S at 10 min after ingestion (*p* < 0.05). For G, the concentration at 60 min was lower than at 20 and 30 min (*p* < 0.05). No other differences were observed over time or between trials at the different time-points. Incremental AUC values were −1856.2 ± 2166.8, 59,342.5 ± 55,279.2, 6510.6 ± 3449.0, 37,052.1 ± 25,605.6, and 39,270.8 ± 33,159.7 pg/mL 1 h, for W, G, F, S, and C, respectively. Incremental AUC was greater for F than W (*p* = 0.028).

Baseline serum GIP concentrations (Table 2) were not different between trials (*p* = 0.246). Effects of trial (*p* < 0.001), time (*p* < 0.001), and interaction (*p* < 0.001), were present for serum GIP concentration (Figure 2b). Concentrations were elevated (*p* < 0.05) from pre-ingestion values for G at all time points (*p* < 0.05). For S, the concentration tended to increase 10 min after ingestion (*p* = 0.052), and were elevated at 20 (*p* = 0.049) and 30 (*p* = 0.036) min after ingestion. This was followed by a decrease at 60 min compared to 20 (*p* = 0.047) and 30 min (*p* = 0.036). For C, concentrations were increased at 10 (*p* = 0.020) and 30 min (*p* = 0.014) compared to baseline. At 10 min after ingestion, concentrations for W and F were lower than G, S, and C (*p* < 0.05), while at 20 min, they were lower than G and S (*p* < 0.05). At 30 min after ingestion, concentrations for W and F were again lower than G, S, and C (*p* < 0.05), and the concentration for G was greater than S (*p* = 0.035). At 60 min after ingestion, the concentration for W was lower than G, S, and C (*p* < 0.05), while the concentration for G was greater than S (*p* = 0.044) and C (*p* = 0.034), and tended to be greater than F (*p* = 0.052). Incremental AUC values were −5.9 ± 111.9, 2224.8 ± 937.2, 50.8 ± 135.0, 1172.3 ± 701.8, and 1252.8 ± 720.7 pg/mL 1 h, for W, G, F, S, and C, respectively. Incremental AUC for G, S, and C, were greater than W (*p* < 0.05), and iAUC for G was greater than F (*p* = 0.014) and S (*p* = 0.033).

Baseline serum GLP-1 concentrations (Table 2) were not different between trials (*p* = 0.092). An effect of time (*p* < 0.001), an interaction effect tending to significance (*p* = 0.078), and no main effect of trial (*p* = 0.354), were present for serum GLP-1 concentration (Figure 2c). GLP-1 concentration was elevated from pre-ingestion values after 10 min (*p* = 0.023), and was lower at 60 min compared to 10 min (*p* = 0.005) for G. Concentrations were also lower at 60 min compared to 10 min for S (*p* = 0.008), and lower at 30 and 60 min compared to 10 min for C (*p* < 0.05). Incremental AUC values for W, G, F, S, and C, were 403.2 ± 655.7, 519.8 ± 345.8, 447.9 ± 390.7, 499.3 ± 574.2, and −131.6 ± 516.3 pg/mL 1 h, respectively. There were no differences in iAUC (*p* = 0.152).

Baseline serum ghrelin concentrations (Table 2) were not different between trials (*p* = 0.066). Effects of trial (*p* = 0.016), time (*p* < 0.001), and interaction (*p* = 0.001), were present for serum ghrelin concentration (Figure 2d). Ghrelin concentration tended to be reduced from pre-ingestion at 20 min for F (*p* = 0.064), and was reduced from pre-ingestion at 60 min (*p* = 0.006). For S and C, reductions from baseline were observed at 30 and 60 min, and 30 min, respectively (*p* < 0.05). Concentrations were also lower at 20 and 30 min, and at 30 min, compared to 10 min for S and C, respectively. No differences over time were present for W and G, although a decrease tending to significance at 60 min compared to 10 min was present for G (*p* = 0.072). At 60 min, W was higher than F (*p* = 0.014). Incremental AUC values were −1892.4 ± 1488, −3028.6 ± 2530.0, −2063.3 ± 1106.9, −3546.1 ± 2073.6, and −2898.5 ± 2007.8 pg/mL 1 h, for W, G, F, S, and C, respectively. No differences were seen in iAUC (*p* = 0.209).

### 3.4. Serum Triglycerides, d-3 Hydroxybutyrate, and NEFA

**Figure 3:**
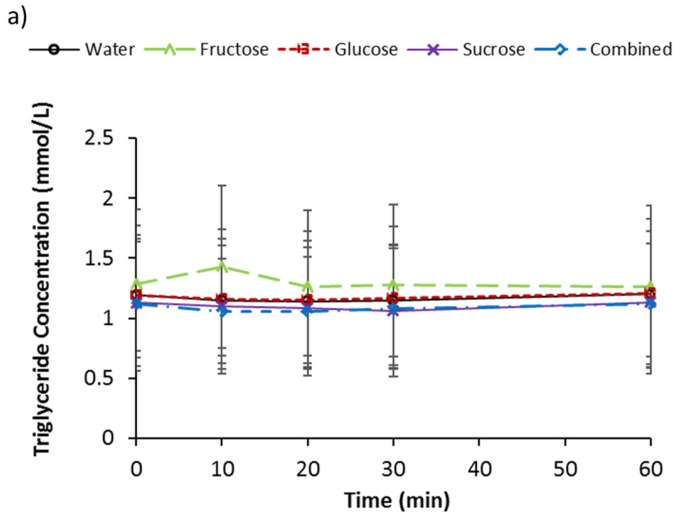

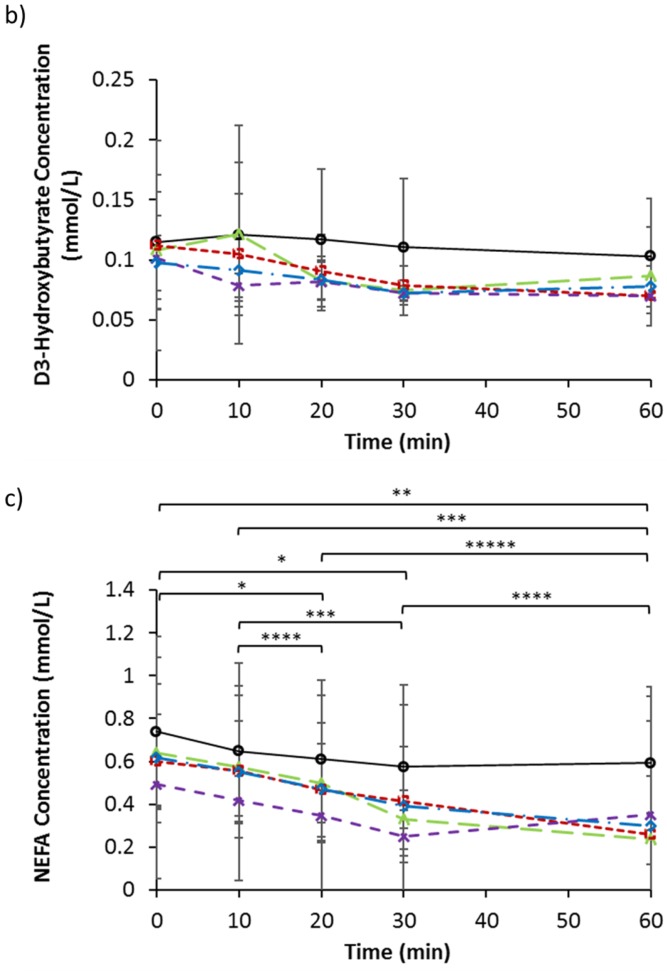
Serum (**a**) triglycerides (**b**) d-3 hydroxybutyrate and (**c**) non esterified fatty acids (NEFA) concentrations at baseline and following ingestion of 595 mL of water (W), 6% fructose (F), 6% glucose (G), 6% sucrose (S) and 6% combined glucose and fructose (C) solutions. * Decrease from baseline for W, G, and C; ** Decrease from baseline for G and C; *** Decrease from 10 min for G and C; **** Decrease from time-point for G; ***** Decrease from time-point for C. All *p* < 0.05. Values are mean ± standard deviation.

Baseline serum triglyceride concentrations (Table 2) were not different between trials (*p* = 0.673). An interaction effect (*p* = 0.032) was indicated for serum triglyceride concentration (Figure 3a). No main effects of trial (*p* = 0.425) or time (*p* = 0.254) were present. A difference at 10 min between trials tended to significance (*p* = 0.099). Differences over time tended to significance for G (*p* = 0.053), S (*p* = 0.092), and C (*p* = 0.073). A difference over time was indicated for W (*p* = 0.039), but no pairwise differences were located, and no change over time was seen for F (*p* = 0.279). Incremental AUC values were −1.81 ± 3.00, −0.65 ± 1.74, 0.54 ± 5.56, −1.97 ± 1.75, and −2.09 ± 3.56 mmol/L 1 h, for W, G, F, S, and C, respectively. No differences were present for iAUC (*p* = 0.534).

Baseline serum d-3 hydroxybutyrate concentrations (Table 2) were not different between trials (*p* = 0.753). No effect of trial (*p* = 0.220), an effect of time tending to significance (*p* = 0.098), and no interaction effect (*p* = 0.891) was present for serum d-3 hydroxybutyrate concentration (Figure 3b). Also, no difference was present for iAUC (*p* = 0.828), where the areas were −0.19 ± 3.05, 1.58 ± 4.30, −1.11 ± 2.89, −1.50 ± 1.72, and −0.99 ± 0.90 mmol/L 1 h, for W, G, F, S, and C, respectively.

Baseline serum NEFA concentrations (Table 2) were not different between trials (*p* = 0.544). No effect of trial (*p* = 0.411) or interaction effect (*p* = 0.431) was present for serum NEFA concentration (Figure 3c), but an effect of time was revealed (*p* = 0.002). Concentrations decreased over time for W, G, and C. For W, the concentrations at 20 and 30 min were lower than baseline (*p* < 0.05). For G, the concentrations at 20, 30, and 60 min were lower than baseline (*p* < 0.01) and 10 min (*p* < 0.05), and in addition, the concentration at 60 min was lower than at 30 min (*p* = 0.040). For C, the concentrations at 20, 30, and 60 min were lower than baseline (*p* < 0.05), the concentrations at 30 and 60 min were lower than at 10 min (*p* < 0.05), and additionally, the concentration at 60 min was lower than at 20 min (*p* = 0.003). No difference was present for iAUC (*p* = 0.512), where the areas were −7.70 ± 5.02, −10.68 ± 3.61, −14.26 ± 13.47, −9.22 ± 9.97, and −11.38 ± 3.96, for W, G, F, S, and C, respectively.

### 3.5. Subjective Measurements of Appetite

**Figure 4:**
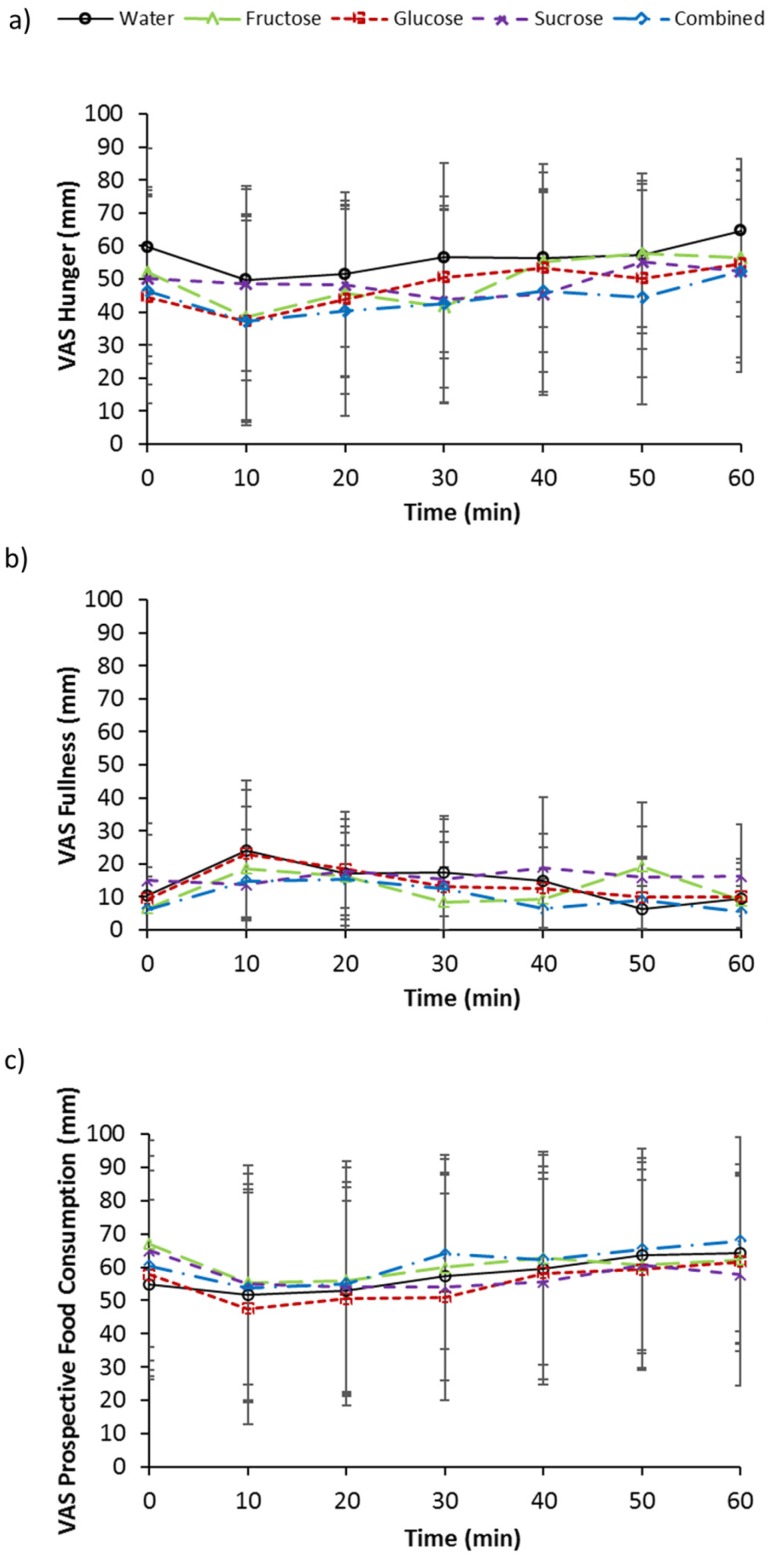
Visual analogue scale (VAS) scores for (**a**) hunger (**b**) fullness and (**c**) prospective food consumption at baseline and following ingestion of 595 mL of water (W), 6% fructose (F), 6% glucose (G), 6% sucrose (S) and 6% combined glucose and fructose (C) solutions. Values are mean ± standard deviation.

A transient pattern of decreased hunger and prospective food consumption ratings occurred at 10 min post drink ingestion followed by a gradual increase thereafter for all trials. Furthermore, consistent with the above, fullness ratings transiently increased at 10 min post ingestion then gradually decreased thereafter. No effect of trial (*p* = 0.337), an effect of time tending to significance (*p* = 0.091), and no interaction effect (*p* = 0.492), were present for hunger ratings (Figure 4a). For fullness ratings, no effects of trial (*p* = 0.455), time (*p* = 0.106), or interaction (*p* = 0.288), were present (Figure 4b). No main effect of trial (*p* = 0.652) or interaction effect (*p* = 0.430) was present for prospective food consumption, but a main effect of time (*p* = 0.001) was seen (Figure 4c). An effect of time was indicated for trial G with 20 min tending to be lower than 60 min (*p* = 0.078). An effect of time also tended to significance for C (*p* = 0.051).

## 4. Discussion

The ingestion of glucose and the ingestion of fructose resulted in respective increases in blood glucose and blood fructose concentration in a dose-related fashion with peak concentrations being attained at 30 min by the glucose alone and fructose alone trials, correspondingly. The similar sucrose and combined solutions resulted in comparable blood glucose and blood fructose concentration responses. Whilst the ingestion of glucose alone resulted in no changes to blood fructose concentration, the ingestion of fructose alone saw a significant increase in blood glucose above water control values at 30 min. This can be explained by the evidence that a moderate amount of fructose undergoes conversion to glucose [19,20]. The ingestion of fructose alone resulted in a significantly lower blood glucose concentration than glucose alone ingestion at 20 min but no significantly lower overall (iAUC) blood glucose response. This result is therefore partially inconsistent with the findings of Kong et al. [3], although the large number of comparisons in this present study may have concealed any statistical difference. A significantly greater blood glucose response was seen with sucrose compared to fructose alone, however, suggesting an interaction of glucose co-ingestion that was not present with the mixed glucose-fructose ingestion. One possibility is the effect of lower osmolality in sucrose resulting in a faster gastric emptying rate and thus a greater increase in the blood glucose concentration. The blood glucose response to different sugars was mirrored by both insulin and GIP responses. However, the pattern of response for GLP-1 did not follow. It is thought that GLP-1 plays a more potent role in glucose-stimulated insulin release, but the results of this study potentially suggest a predominant role of the incretin GIP.

The ingestion of solutions containing fructose resulted in significant increases in blood lactate concentration at a faster rate than the ingestion of glucose alone. This increase in lactate concentration occurred even with the relatively small amount of fructose ingestion (18 g) within the S and C trials. Furthermore, the ingestion of sucrose, and combined glucose and fructose, resulted in similar iAUCs, compared to fructose ingestion alone, despite containing half of the amount of fructose. This may be due to a differential fate of fructose when co-ingested with glucose. The presence of glucose in the ingested solutions may have led to the preferential oxidation of glucose within the Krebs cycle, as well as the conversion to glycogen, thus limiting this pathway for fructose oxidation and resulting in greater lactate production. It is unlikely that this was due to reduced insulin action, which is reported to result in less pyruvate entering the mitochondria for oxidation and thus causing a corresponding increase of anaerobic metabolism to lactate [21], because insulin secretion following both S and C were pronounced in comparison to fructose. Another potential explanation is related to the observations that fructose absorption is augmented when ingested with glucose [22]. However, it is unlikely that the observed results were due to a greater or more efficient absorption of fructose when co-ingested with glucose, as serum fructose concentration increased significantly from baseline following fructose alone ingestion but not for the fructose-glucose solutions.

The ingestion of all four sugar solutions resulted in similar acylated ghrelin suppression, unlike the finding by Teff et al. [6] that fructose ingestion results in less suppression following fructose ingestion when compared to glucose ingestion. Furthermore, little difference between sugars was observed for GLP-1 response. This is in contrast to previous findings by Kong et al. [3] and Kuhre et al. [23] where participants were fed 75 g of sugar in both studies. Although it is noted that a potential limitation of the present study is that we measured total GLP-1, and not the specific active form GLP-1^7–36^, the reported difference seen by Kuhre et al. [23] was also for total GLP-1, indicating the contrasting findings are likely due to the lower amount of sugar ingestion (36 g) in the present study. However, a marked difference between sugars was seen with GIP responses. The ingestion of fructose induced virtually no GIP response in contrast to the other sugar solutions, and was comparable to the effects of water. Although insulin concentrations significantly increased and then decreased following glucose and sucrose ingestion, whilst no significant changes over time was observed for fructose ingestion, there were no significant differences detected between sugars at different time-points or for the overall (iAUC) response. The insulin results are therefore inconsistent with those of previous studies that have shown significantly lower responses following fructose ingestion compared to glucose ingestion [3,4,5]. This may be due to the large number of comparisons in the present study masking any differences. Alternatively, this may have been due to the large standard deviations observed. The large inter-individual variability may be due to the differences in the body mass index of the participants, which ranged from normal to obese classifications. The range of participants utilised in this study is a limitation as insulin and metabolic responses may differ in participants with different levels of adiposity. However, as this study utilised a repeated measures design, each participant acted as their own control. In line with the absence of response differences in the gut-derived appetite hormones ghrelin and GLP-1, no subsequent difference or effect of sugar ingestion was observed for any of the appetite ratings.

Triglyceride concentrations were unchanged following ingestion of the sugar solutions, and no difference was found between trials, suggesting that the acute ingestion of simple sugars in typical amounts does not result in immediate increases in the rate of de novo lipogenesis. However, it may be that the 60 min postprandial measurement period in the present study was not long enough to detect any changes, as triglyceride concentrations have been shown to be significantly elevated 2–3 h after fructose ingestion [24]. The one-hour postprandial measurement period was selected based on the main responses of blood glucose, GLP-1, and insulin, occurring within 60 min after the ingestion of a much larger bolus of fructose (75 g) in studies with two-hour [3] and four-hour [4] measurement periods. In addition, whilst significant decreases in NEFA concentrations for W, G, and C trials were observed over time, no differences in NEFA or d-3-hydroxybutyrate concentration suppression was seen between sugar trials, indicating that the ingestion of the different sugars resulted in similar reductions in lipolysis and NEFA metabolism. This is consistent with the studies by Ngo Sock et al. [16], Teff et al. [5], and Teff et al. [6]. For the trials involving glucose ingestion, this is consistent with the elevation and action of insulin. However, this is unlikely to be the mechanism for reduced NEFA concentrations following fructose ingestion as insulin secretion was relatively unchanged. Instead, the mechanism relating to this may be explained by the increased lactate production seen with fructose ingestion. Lactate has been shown to inhibit lipolysis in adipocytes [25].

Whilst the participants were asked to record their food intake and physical activity in the 24 h prior to their first experimental trial, for the purpose of standardisation by repeating these in subsequent trials, no dietary information was collected on the habitual consumption of sugar-sweetened beverages. Long periods of high fructose intake at 25% of the energy requirements can alter glucose and insulin responses within ten weeks, and markers of lipid metabolism within two weeks [13,14]. However, during the relatively short period of study, it would have been unlikely that any participants had such a large change in their habitual diet in the present study. Furthermore, as it was a repeated measures design, each participant acted as their own control so this would not affect the conclusions made. A limitation of the current study is the generalisability of these novel results on the effect of ingestion of simple sugars in amounts reflective of typical consumption, as only healthy men were studied in the present study. Hormonal and metabolic responses to simple sugar ingestion in women may differ, and future research in this area should explore whether there are any sex differences, in addition to the responses of those who are obese or who have other metabolic disorders.

## 5. Conclusions

The acute ingestion of simple sugars in typical amounts induced marked differences in the circulating GIP response, and blood glucose and fructose responses, but not acylated ghrelin, total GLP-1, or insulin responses. No effects on appetite scores were seen as a result. The acute ingestion of a solution containing typical amounts of sugar does not result in significantly increased triglyceride synthesis over the postprandial period investigated. Furthermore, no differences between sugars in these smaller quantities were seen for lipolysis and NEFA metabolism suppression but fructose ingestion results in significantly increased lactate production that is augmented with glucose co-ingestion.

## Figures and Tables

**Table 1:** Pre-trial body mass and urine characteristics pre- and post-trial.

	Water		Glucose		Fructose		Sucrose		Combined	
	Mean	SD	Mean	SD	Mean	SD	Mean	SD	Mean	SD
Body mass (kg)	81.52	12.03	81.84	11.77	81.80	12.31	81.93	12.06	81.54	12.42
Pre urine volume (mL)	174	114	199	178	143	136	164	120	179	197
Post urine volume (mL)	613	268	639	226	411	254	577	400	596	331
Pre urine osmolality (mOsmol/kg)	461	232	375	224	431	174	593	309	465	260
Post urine osmolality (mOsmol/kg)	161 ^a^	101	137 ^a^	59	233 ^a^	148	185 ^a^	157	269	299

^a^ Significantly lower than pre urine osmolality (*p* < 0.05). Values are means and standard deviations (SD).

**Table 2:** Baseline concentrations for blood serum measures.

	Water		Glucose		Fructose		Sucrose		Combined	
	Mean	SD	Mean	SD	Mean	SD	Mean	SD	Mean	SD
Glucose (mmol/L)	5.15	0.39	5.11	0.28	5.27	0.21	5.25	0.18	5.12	0.26
Fructose (µM/L)	67.34	19.59	60.70	41.81	51.75	37.31	57.95	36.73	64.62	40.65
Lactate (mmol/L)	0.93	0.27	1.13	0.30	0.91	0.23	0.94	0.18	0.88	0.24
Insulin (pg/mL)	191.4	88.5	216.9	163.1	192.1	102.3	172.4	103.4	177.7	89.4
GIP (pg/mL)	8.81	3.33	12.67	7.71	9.31	5.26	12.12	8.82	13.15	7.20
GLP-1 (pg/mL)	58.4	6.3	61.7	4.2	64.1	12.5	62.9	11.4	69.2	14.2
Ghrelin (pg/mL)	232.1	79.6	200.9	80.2	189.0	68.8	220.7	84.2	189.9	65.1
Triglycerides (mmol/L)	1.20	0.47	1.19	0.59	1.29	0.62	1.13	0.56	1.13	0.52
d-3 Hydroxybutyrate (mmol/L)	0.12	0.06	0.11	0.09	0.11	0.05	0.10	0.03	0.10	0.02
NEFA (mmol/L)	0.74	0.35	0.60	0.22	0.64	0.32	0.50	0.12	0.62	0.56

Values are means and standard deviations (SD).
